# Re-treatment of relapse in elderly AML with time-limited venetoclax-based regimen: a case report and literature review

**DOI:** 10.1007/s00277-026-06781-z

**Published:** 2026-02-17

**Authors:** Jun Yen Ng, Veena Gullapalli, Jad Othman, Dipti Talaulikar

**Affiliations:** 1 Department of Haematology, ACT Pathology, Canberra Hospital, Canberra, Australia; 2 School of Medicine and Psychology, College of Science and Medicine, Australian National University, Canberra, Australia; 3 Institute of Haematology, Royal Prince Alfred Hospital, Camperdown, NSW Australia; 4 Prince of Wales Clinical School, University of New South Wales, Sydney, NSW Australia; 5 Department of Haematology, Royal North Shore Hospital, St Leonards, NSW Australia; 6 Department of Diagnostic Genomics, ACT Pathology, Canberra, Australia

**Keywords:** Acute myeloid leukaemia, Venetoclax, NPM1

## Abstract

Venetoclax and hypomethylating agent combination therapy is now the standard of care in acute myeloid leukemia (AML) for patients ineligible for intensive therapy, including the elderly; however, venetoclax and low-dose cytarabine remain a viable option for select patients. Relapse in this cohort remains a significant challenge, with a poor prognosis and an unmet need for further treatment options. This case discusses an elderly patient with *NPM1-*mutated AML successfully retreated with time-limited venetoclax and low-dose cytarabine at relapse. The sustained response observed contributes to the limited literature on the efficacy of re-treatment with venetoclax-based therapy in this setting. However, prospective data are required to assess the efficacy and safety of this strategy as well as to establish the role of molecular monitoring. The role of time-limited venetoclax therapy for preventing treatment resistance and limiting treatment-related adverse effects remains an important question in the cohort ineligible for intensive therapies, given the poor prognosis and limited options of relapsed/refractory AML post-venetoclax and hypomethylating agent therapy.

## Case presentation

Dear editor,

A 70-year-old male was investigated for cytopenia and macrocytosis in 2016. Medical history included idiopathic dilated cardiomyopathy and chronic inflammatory demyelinating polyneuropathy managed with monthly intravenous immunoglobulin (IVIg). Bone marrow examination (BME) diagnosed Myelodysplastic syndrome (MDS), subtype Refractory cytopenia with multilineage dysplasia (RCMD) (World Health Organization (WHO) classification, 2008) with 2.6% blasts and a normal karyotype. The International Prognostic Scoring System (IPSS) risk category was intermediate-1, and the Revised IPSS (IPSS-R) risk category was low [1]. Next-generation sequencing (NGS) was not performed.

**Fig. 1:**
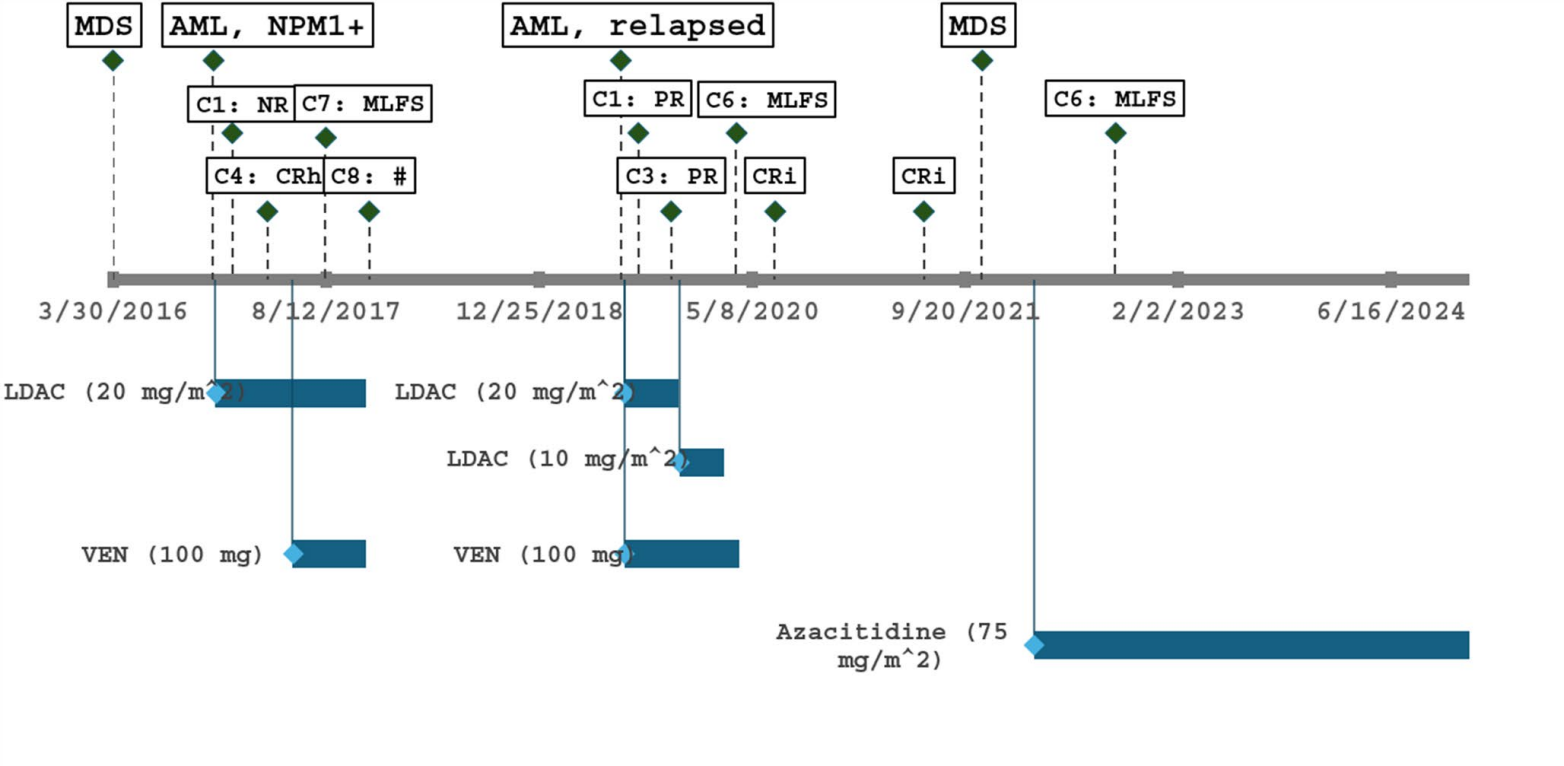
Timeline of clinical course and treatment of MDS and AML * AML* acute myeloid leukemia, *C* cycle, *CR* complete remission, *CRh* complete remission with partial hematologic recovery, *CRi* complete remission with incomplete hematologic recovery, *LDAC* low-dose cytarabine, *MDS* Myelodysplastic neoplasm, *MLFS* morphologic leukemia-free state, *NR* no response, *PR* partial remission, *VEN* venetoclax ◆findings from bone marrow examination response assessments performed after the indicated cycle numbers, where relevant #: Bone marrow examination shortly after acute decompensated heart failure showed left-shifting with approximately 5% of blasts. Created using template by Wittwer, JW (4)

Eight months later, repeat BME for progressive cytopenia diagnosed transformation to acute myeloid leukemia (AML) with *NPM1* mutation (WHO classification, 2022) [2]. The karyotype remained normal, and NGS demonstrated additional *SRSF2* and *TET2* mutations. *DDX41* was not tested as part of the panel. The patient was classified as favorable-risk disease according to the 2024 European Leukemia Net (ELN) risk stratification [3]. He received eight cycles of low-dose cytarabine (LDAC; 20 mg/m^2^, days 1 to 10), and venetoclax (VEN; 100 mg, days 1 to 14) was added after the first five cycles (Fig. 1). Complete remission with incomplete hematologic recovery (CRi) was achieved, and the *NPM1* and *SRSF2* mutations became undetectable at a 2% variant allele frequency. Recurrent febrile neutropenia, dental abscesses and worsening cardiac failure with acute decompensation required cessation of treatment for AML. He remained in remission without further therapy for 19 months. He was considered unsuitable for an allogeneic stem cell transplant.

**Fig. 2:**
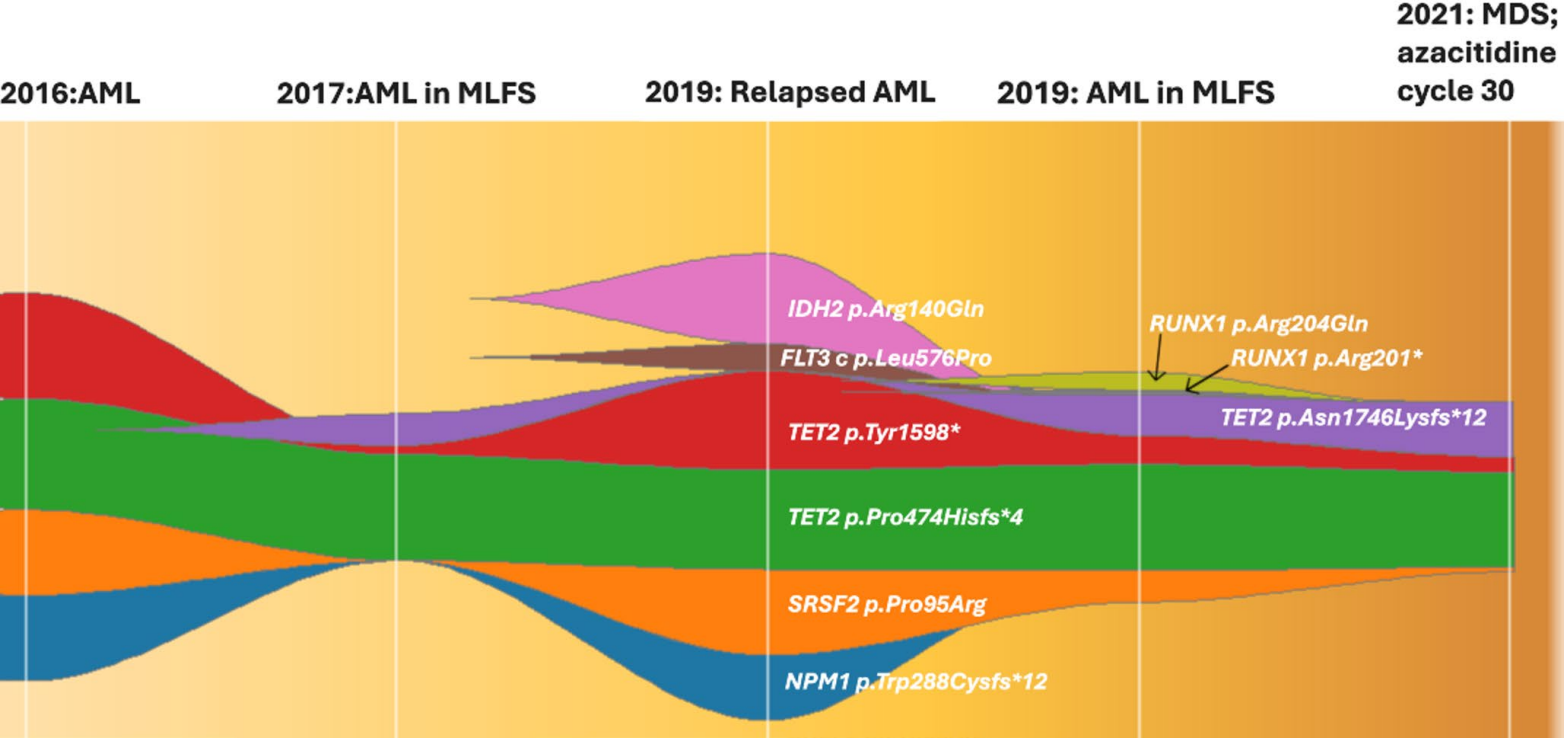
VAF plot from 2016 to 2021. *AML* acute myeloid leukemia, *MDS* Myelodysplastic neoplasm, *MLFS* morphologic leukemia-free state, *VAF* variant allelic frequency

Re-treatment commenced in 2019 for relapsed AML with recurrence of the *NPM1* and *SRSF2* clone (Fig. 2). Additional *FLT3* and *IDH2* mutations were also identified (see Table 1.); however, targeted therapies were not accessible. VEN (100 mg daily, days 1 to 14) and LDAC (20 mg/m^2^, days 1 to 10) were initiated, and after three cycles, LDAC was reduced to 10 mg/m^2^, days 1 to 10, for a further three cycles secondary to ocular toxicity and worsening neuropathy related to pre-existing CIDP (see Fig. 2). Treatment was complicated by febrile neutropenia and Campylobacter gastroenteritis. The best response achieved by the patient was CRi, and the *NPM1*, *FLT3*, and *IDH2* clones became undetectable by NGS. However, a *SRSF2* clone remained. Transfusion-independent cytopenia persisted without evidence of relapse on repeat BMEs (Table 1.). Treatment was ceased after six cycles.

**Table 1.**
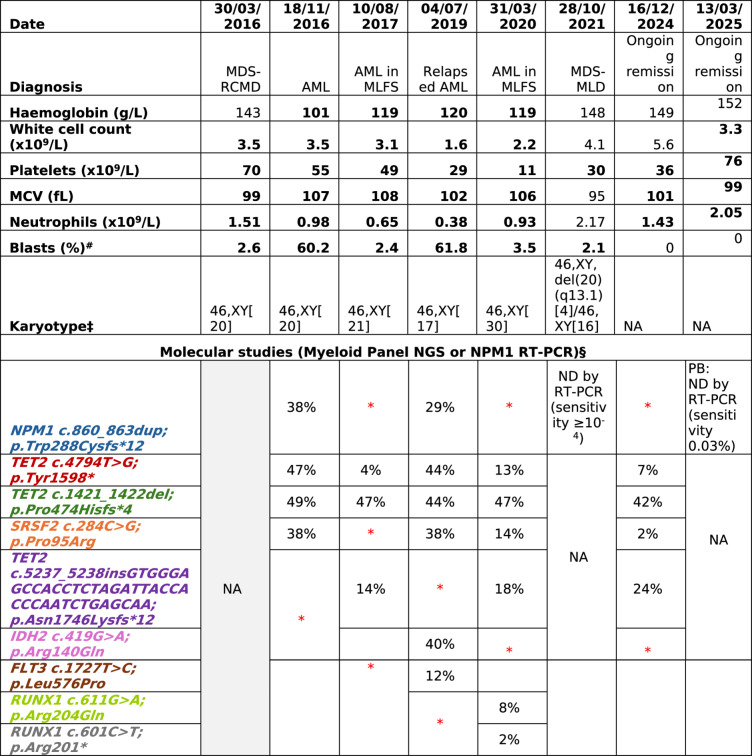
Peripheral blood and bone marrow results from 2016 to 2025

* AML* acute myeloid leukemia,*CR* complete remission, *CRh* complete remission with partial hematologic recovery, *CRi* complete remission with incomplete hematologic recovery, *MCV* mean corpuscular volume, *MDS* Myelodysplastic neoplasm, *MLD* multilineage dysplasia, *MLFS* morphologic leukemia-free state, *MRD* measurable residual disease,*NA* not available, *ND* not detected, *NGS* next-generation sequencing,*NR* no response, *PB* peripheral blood, *PR* partial remission, *RCMD* refractory cytopenia with multilineage dysplasia, *RT-PCR* reverse transcription polymerase chain reaction # Blast enumeration on morphological assessment of bone marrow aspirate unless stated otherwise ‡Karyotype, NGS, and MRD performed on bone marrow specimens unless stated otherwise * Not detected at a detection limit of approximately 4-5%§ Percentages correspond to the VAFs in NGS ^ Figure created using the Fishplot package on R.(5) The variants, VAF changes, and date of each test correspond to Table 1.

In 2021, repeat BME for worsening neutropenia diagnosed MDS with multi-lineage dysplasia (MLD) and a karyotype showing del(20)(q13.1) in 20% of cells. The IPSS score was calculated to be 0.5. NGS was not performed at this point; however, *NPM1* measurable residual disease (MRD) testing with a sensitivity of 10^− 5^ was negative. Azacitidine (75 mg/m^2^, days 1–7) was commenced, and to date, the patient has received the 33 cycles, and therapy remains ongoing. The AML and MDS remain in remission nine years after the initial diagnosis, with persistent *NPM1* MRD negativity, transfusion independence and repeated BME demonstrating normal hematopoiesis.

VEN is a selective inhibitor of B-cell leukemia/lymphoma-2 (BCL2) protein. The efficacy and safety of VEN-based regimens in elderly patients with treatment-naive AML ineligible for intensive therapy has been demonstrated [6, 7]. While VEN and hypomethylating agent (HMA) combination therapy is now the standard of care in AML patients ineligible for intensive therapy, VEN and LDAC remain a viable option for select patients [18]. VIALE-A and VIALE-Cs used indefinite therapy till disease relapse or treatment intolerance, but neither was published when AML was diagnosed in our patient. VEN was accessed through his insurance company after five cycles of LDAC based on expert advice. Treatment was ceased after three cycles of combination therapy because of intercurrent illness and lack of evidence on the duration of therapy.

Relapse in elderly AML remains a significant challenge. In the VIALE-A study, which studied an elderly cohort (age > 75) with previously untreated AML, 52% and 74% of participants in the VEN + azacitidine and azacitidine + placebo arms developed progressive disease (PD) or morphologic relapse (MR) after a median follow-up of 43.2 months, respectively [8]. In the VIALE-C study, as compared to patients treated with LDAC + placebo, those treated with VEN + LDAC had a longer median event-free survival (4.7 vs. 2 months; *P* = 0.002) and a higher CR/CRi rate (48.3% vs. 13.2%) with a median duration of response of 11.8 months after a median follow-up of 34.7 months [7, 9]. 

The prognosis of relapsed/refractory (R/R) AML post-VEN-based regimens is poor, with a median OS of 2.4–6.8 months [8, 10, 11]. Treatment options are limited in patients ineligible for allogeneic hematopoietic stem cell transplant (alloHCT) or intensive chemotherapy. In a retrospective single-centre study of 41 patients with R/R AML after frontline VEN and HMA therapy, in comparison to untreated patients (*n* = 17), salvage therapy (*n* = 24) resulted in minimal improvement in OS compared to untreated patients (2.9 vs. 1.3 months, HR 0.41, 95% CI 0.19–0.88; *P* = 0.003) [10]. Due to the limited success expected with other treatment strategies and the accumulation of evidence for continuous VEN therapy in VIALE-A and VIALE-C, it was decided to recommence the patient on VEN and LDAC. The patient had previously not demonstrated disease progression with VEN therapy.

There is limited data regarding the re-treatment of R/R AML with VEN-based regimens. In a retrospective single-centre study, 33% (5/15) of patients with AML responded to re-treatment with VEN and HMA at relapse, with two patients attaining a negative MRD. The median age was 66 (19–81) years, 11 (73%) patients had de novo AML, and 8 (53%) patients had high-risk disease according to 2017 ELN risk stratification. Fourteen (93%) patients received decitabine, and one patient received azacitidine during initial treatment, with a median of 2 cycles of treatment. The median duration between discontinuation of initial therapy and re-treatment was 224 days (73–407 days) [12]. Mortality during the first 30 days of re-treatment was 20%. One patient who responded to treatment underwent a second alloHCT. The median overall survival (OS) for all patients was 205 days after re-treatment, whereas the median leukemia-free survival for patients who responded was 315 days [12]. Furthermore, Tamellini et al. reported a patient who relapsed 12 months after achieving CR with intensive anthracycline-based therapy and achieved a sustained CR after 3 cycles of VEN-HMA. The patient declined further therapy and relapsed after 12 months. Retreatment with VEN—azacitidine for 13 cycles achieved a CRi [13]. Thus, there is insufficient evidence of the efficacy of re-treatment in relapse after treatment interruption or discontinuation.

VEN resistance in AML occurs by several mechanisms, particularly the upregulation of Mcl-1, an anti-apoptotic protein in the BCL2 family [14]. We hypothesize that the limited initial treatment duration may have reduced the risk of resistance and enabled disease sensitivity in re-treatment. Given that retreatment was effective in our case, it raises the question of whether treatment in the elderly should be time-limited if CR/CRi is achieved to limit toxicity and prevent the development of treatment resistance. Othman et al. demonstrated that transplant-ineligible patients treated with VEN and LDAC or azacitidine achieved prolonged treatment-free remission after cessation of therapy if MRD is achieved within four cycles of treatment [15]. To the authors’ knowledge, VEN and LDAC time-limited re-treatment for relapsed AML, including *NPM1*-mutated cases and MRD responses with re-treatment in this cohort, has not been reported. Prospective studies are needed to determine the efficacy of time-limited therapy and the re-initiation of VEN-based therapy in morphological or measurable residual disease relapse. In particular, patients with similar characteristics to this case, including ELN-defined favourable-risk disease and absence of VEN-refractoriness, may benefit from this approach.

Interestingly, the patient in remission demonstrated MDS with persistence of original *TET2* and *SRSF2* mutations and acquisition of further *TET2* and *RUNX1* mutations (see Fig. 1). Thus, it supports that *NPM1* is a strong leukemogenic driver, and the absence of *NPM1* MRD could indicate remission from the leukemic clone [16]. Furthermore, the prolonged exposure to azacitidine resulted in the re-programming of the MDS clone to induce normal hematopoiesis [19] and may have prevented the recurrence of the leukemic clone [17]. Alternatively, the MDS clone may have emerged post-cytotoxic therapy, with the acquisition of del (20) on karyotype.

This case contributes to the limited literature on the efficacy of re-treatment with VEN-based therapy in elderly patients with relapsed AML, the clonal evolution of disease recurrence, and long-term outcomes in re-treatment. Further data from prospective studies assessing the efficacy and safety of VEN re-treatment and establishing the role of molecular monitoring are required. The role of time-limited VEN therapy as a method of preventing treatment resistance and limiting treatment-related toxicity remains an important question in the transplant-ineligible cohort, given the poor prognosis and limited options of R/R AML post-VEN and HMA therapy.

## Data Availability

Enquiries about data access should be made to the corresponding author.
